# Supplementary material for: Prioritizing supports and services to help older adults age in place: A Delphi study comparing the perspectives of family/friend care partners and healthcare stakeholders
Source: PLoS One. 2021 Nov 9;16(11):e0259387. doi: 10.1371/journal.pone.0259387 (PMC8577765; doi:10.1371/journal.pone.0259387)
Supplement: S1 File — (PDF) [file pone.0259387.s003.pdf]

SPOR Delphi Survey

Round 1

Goal

Thank-you for agreeing to participate in our survey. This survey is being conducted by a group of researchers, healthcare planners and community advisors from Manitoba and Alberta. **Our goal** is to identify factors that you feel are most important to help older adults (age 65+) live successfully in the community (i.e., to be independent, safe, and have as high a quality of life as possible). Our results will be used to help planners identify the types of programs and services that are needed to help achieve this goal.

This survey should take you about 30 minutes to complete, and we have provided a set of directions to help you complete it. **It is important that you complete this survey within two weeks of receiving it**, so that we can compare everyone's results and prepare for the next survey round in a timely manner. You will receive a reminder email 7 days from now if we have not received your completed survey by this time.

Please contact Megan Campbell (Study Coordinator) at XXX-XXX-XXXX or by email XXXXXXXXXX@XXXXXXXXXX.ca if you have any questions about the survey, or about any other information that we have provided.

Directions

Directions to Complete This Survey

1. Please complete the consent and participant information sections.

2. We have included 23 factors in this survey. **We are asking you to score each factor according to how important you think this is for helping older people to live in the community**. Please score each factor using a number between 1 and 10 keeping the following in mind:

   <u>#1 represents a score of "Not important"</u> - By providing this score, you are saying that a factor does not help older adults to live successfully in the community.

   <u>#5 represents a score of "Somewhat important"</u> - The factor may help SOME older adults to live successfully in the community.

   <u>#7 represents a score of "Quite Important"</u> - The factor helps most older adults to live successfully in the community, but there are more important things to consider.

   <u>#8 represents a score of "Very Important"</u> - The factor is amongst the top 5 or 6 things needed to help most older adults live successfully in the community.

   <u>#10 represents a score of "Absolutely Critical"</u> - The factor is amongst the top 1 or 2 things that you feel are needed to help most older adults live successfully in the community.

3. At the end of the survey, you can provide additional factors that you feel are missing from our list. We will include your suggestions in the next survey round. We will ask you to complete modified versions of this survey, three times. We may also ask additional questions in later survey rounds.

Our goal is to have you choose a smaller number of factors that you feel are important to help older adults live successfully in the community. Please only rate factors as "very important" (score of 8 or higher) if they are amongst the top 5 or 6 items that you would choose.

We have provided space for you to briefly rationalize your score. You do not need to justify each of your responses, but we suggest doing so for factors that you feel most strongly about (e.g., score 8 and higher).

EXAMPLE:
0. Having a balanced diet

1	2	3	4	5	6	7	8	9	10
○	○	○	○	○	○	◉	○	○	○

Comments

While important, most older adults are able to eat a balanced diet without supports.

Consent Section

All people who wish to complete the Survey MUST complete this section

Your participation in this research study is completely voluntary and confidential (no other participants will see your individual responses). All survey data will be de-identified (i.e., prior to storing study results, your name will be separated from your survey responses) and stored on a password protected computer.

You may decide to withdraw from the study at any time, in which case we will not include your results in subsequent analysis of the data. To withdraw from this study, please contact the study coordinator (Megan) by email at XXXXX@XXXXXXX.ca.

This study is funded by the Canadian Institutes of Health Research and has been approved by the University of Manitoba Health Research Ethics Board, the University of Alberta Health Research Ethics Board, the Winnipeg Regional Health Authority and Alberta Health Services.

* **Important:** Please only check this box if you consent to participate in this study. Your consent indicates that you agree to the following:

- We will include your results in our study
- We can send subsequent survey rounds to you
- You will be involved for the duration of the study (note the anticipated end date is late September, pending how quickly people complete the survey)

☐ I Consent

Participant Information Section

* Age:
(years)

* Sex:

- Male
- Female

* Province:

- Alberta
- Manitoba

Expertise Questions

* 1. What is your current job title?

* 2. For how long have you been in your current position?
(years/months)

* 3. For how long in your career have you focused on services for older adults?
(years/months)

* 4. What is the highest degree or level of school you have completed?
(If currently enrolled, select highest degree received)

- a. Some high school, no diploma
- b. High school graduate, diploma or the equivalent (for example: GED)
- c. Some college credit, no degree
- d. Trade/technical/vocational training

- ○ e. Bachelor’s degree – please provide type: [ ]
- ○ f. Master’s degree – please provide type: [ ]
- ○ g. Professional degree (e.g., nurse, pharmacists, social worker, physician) – please provide type: [ ]
- ○ h. Doctorate (PhD) degree – please provide type: [ ]
- ○ i. Other – please provide details: [ ]

Questions

Please rate the importance of each of the following 23 factors in helping older adults live successfully in the community (i.e., to be independent, safe, and have as high a quality of life as possible), keeping the following in mind:

#1 represents a score of "Not important" - By providing this score, you are saying that a factor does not help older adults to live successfully in the community.

#5 represents a score of "Somewhat important" - The factor may help SOME older adults to live successfully in the community.

#7 represents a score of "Quite Important" - The factor helps most older adults to live successfully in the community, but there are more important things to consider.

#8 represents a score of "Very Important" - The factor is amongst the top 5 or 6 things needed to help most older adults live successfully in the community.

#10 represents a score of "Absolutely Critical" - The factor is amongst the top 1 or 2 things that you feel are needed to help most older adults live successfully in the community.

*Please limit the number of factors rated with the highest scores.

Comments - You can use this space to briefly comment on your rating. You do not need to justify each of your responses, but we suggest doing so for factors that you feel most strongly about.

---

1. Keeping physically active

1 2 3 4 5 6 7 8 9

10

Comments

2. Being continent (with or without the use of continence aids)

1 2 3 4 5 6 7 8 9

10

Comments

3. Having medical professionals (e.g., nurse practitioners, pharmacists) who regularly check the # and type of

Comments

medications people are taking

1 2 3 4 5 6 7 8 9

10

4. Not having significant behavioural or mental health disorders

Comments

1 2 3 4 5 6 7 8 9

10

5. Thinking of oneself as healthy

Comments

1 2 3 4 5 6 7 8 9

10

6. Maintaining a positive attitude, having a high self-esteem and/or sense of personal identity

Comments

1 2 3 4 5 6 7 8 9

10

7. Keeping mentally active

Comments

1 2 3 4 5 6 7 8 9

10

8. Being someone who prepares and

Comments

plans for the future (e.g., participates in health promotion activities, plans financially for the future, develops new skills)

1 2 3 4 5 6 7 8 9

10

9. Living in a safe home environment (e.g., with enough safety aids and equipment)

1 2 3 4 5 6 7 8 9

10

Comments

10. Having a home layout that is appropriate (e.g., the absence of stairs)

1 2 3 4 5 6 7 8 9

10

Comments

11. Having enough money to afford to stay successfully in the community

1 2 3 4 5 6 7 8 9

10

Comments

Reminder:

Rate the importance of each factor in helping older adults live successfully in the community (i.e., to be independent, safe, and have as high a quality of life as possible).

#1 represents a score of “Not important”
#5 represents a score of “Somewhat important”
#7 represents a score of “Quite Important"
#8 represents a score of “Very Important”
#10 represents a score of “Absolutely Critical”

*Please limit the number of factors rated with the highest scores.

---

12. Having strong relationships and links with family, friends, and the community

1 2 3 4 5 6 7 8 9

10

Comments

13. Having accessible and affordable community-based services (e.g., adult education, recreation and support programs)

1 2 3 4 5 6 7 8 9

10

Comments

14. Having a resource (e.g., information call centre) that helps people make informed choices about health care services that are available to them

1 2 3 4 5 6 7 8 9

10

Comments

15. Having public transportation that is affordable, reliable and accessible

1 2 3 4 5 6 7 8 9

Comments

10

16. Having formal healthcare providers (e.g., physicians, home care workers) who are aware of community-based services

1 2 3 4 5 6 7 8 9

10

Comments

17. Having training & education programs for informal caregivers

1 2 3 4 5 6 7 8 9

10

Comments

18. Having good communication between informal & formal caregivers

1 2 3 4 5 6 7 8 9

10

Comments

19. Having coordinated care between all types of formal health care providers (e.g., physicians, home care workers, social workers).

1 2 3 4 5 6 7 8 9

Comments

10

20. Having physicians who provide house-calls & home visits

1 2 3 4 5 6 7 8 9

10

Comments

21. Having access to affordable housing

1 2 3 4 5 6 7 8 9

10

Comments

22. Having policies that allow people to reside in the community with an acceptable level of risk

1 2 3 4 5 6 7 8 9

10

Comments

23. Having access to funds that help people purchase assistive technology (e.g., motorized wheelchairs) and/or to modify their home (e.g., put in a wheelchair ramp).

1 2 3 4 5 6 7 8 9

10

Comments

Below, you can provide additional factors that you feel are missing from our list

24. Additional Factor #1 (Please Describe)

Rate the importance of your new factor

1 2 3 4 5 6 7 8 9

10

Comments

25. Additional Factor #2 (Please Describe)

Rate the importance of your new factor

1 2 3 4 5 6 7 8 9

10

Comments

26. Additional Factor #3 (Please Describe)

Rate the importance of your new factor

1 2 3 4 5 6 7 8 9

10

Comments

27. Additional Factor #4 (Please Describe)

Rate the importance of your new factor

1 2 3 4 5 6 7 8 9

10

Comments

28. Additional Factor #5 (Please Describe)

Rate the importance of your new factor

1 2 3 4 5 6 7 8 9

10

Comments

Powered by SimpleSurvey
